# Social dominance orientation as an obstacle to intergroup apology

**DOI:** 10.1371/journal.pone.0211379

**Published:** 2019-01-25

**Authors:** Nobuhiro Mifune, Kazunori Inamasu, Shoko Kohama, Yohsuke Ohtsubo, Atsushi Tago

**Affiliations:** 1 School of Economics & Management, Kochi University of Technology, Kochi, Kochi, Japan; 2 School of Sociology, Kwansei Gakuin University, Nishinomiya, Hyogo, Japan; 3 Public Policy School, Hokkaido University, Sapporo, Hokkaido, Japan; 4 Department of Psychology, Graduate School of Humanities, Kobe University, Kobe, Hyogo, Japan; 5 School of Political Science and Economics, Waseda University, Shinjuku, Tokyo, Japan; 6 Global Fellow, Peace Research Institute Oslo (PRIO), Oslo, Norway; Ghent University, BELGIUM

## Abstract

Social Dominance Orientation (SDO) has engaged the interest of social and personality psychologists as it has deep implications for the psychology of intergroup conflict, particularly regarding factors such as prejudice and discrimination, as well as international conflict resolution. Nevertheless, few studies have directly assessed how SDO relates to intergroup reconciliation. This study (effective *N* = 819) measured participants’ SDO along with their attitudes toward various governmental apologies to test the hypothesis that SDO is associated with unwillingness to issue intergroup apologies. The results showed that SDO was negatively correlated with supportive attitudes toward government-issued international apologies. This negative correlation remained intact after controlling for the effects of political conservatism and militarism.

## Introduction

Conflicts between various social categories and groups, such as nations, ethnic groups, and races, are widely observed in the contemporary world. Mechanisms underlying the emergence and persistence of intergroup conflicts have been studied in several of the social sciences, such as political science, sociology, and economics, as well as social psychology [1, 2, 3]. Although social psychology tends to focus on situational factors [4, 5], there are studies that have identified individual differences that intensify intergroup conflict [6, 7, 8, 9]. In light of these individual difference variables, the current study investigates the way social dominance orientation (SDO; [10]) relates to the process of resolving intergroup conflict, particularly the relation between SDO and unwillingness to apologize.

SDO, or “individuals’ desires for group-based dominance and inequality” [11], has been found to positively correlate with prejudicial or discriminatory attitudes towards various social categories [10, 11, 12, 13]. For example, SDO is correlated with negative views toward African Americans among Caucasian Americans [14], hostile sexism [15, 16], and negative attitudes toward immigrants among Canadians [17]. These correlations are observed not only in Western countries but also in Eastern countries, such as China and Taiwan [18]. Studies conducted in Japan also showed that it correlates with discriminatory attitudes toward Korean residents in Japan and xenophobic attitudes among Japanese [19, 20]. Despite the wealth of literature suggesting a close association between SDO and pro-conflict attitudes and behaviors, such as prejudice and discrimination, few studies have investigated the relation between SDO and pro-reconciliation attitudes [21], such as forgivingness and willingness to apologize.

Apologies are one of the major factors facilitating interpersonal forgiveness [22]. Hornsey et al. [23] tested the prediction that SDO would have a negative effect on willingness to apologize. In their study, participants’ willingness to apologize was assessed by the Proclivity to Apologize Measure (PAM; [24]). They confirmed that SDO is negatively associated with an individual’s relatively stable tendency to apologize. However, the PAM comprises context-free items (e.g., “To avoid feeling incompetent, I tend not to apologize”) and interpersonal items (e.g., “I tend to downplay my wrongdoings to the other person, rather than apologize”). One might expect that individuals high in SDO are also reluctant to issue intergroup/political apologies because the SDO is conceptually tied to concerns about superiority among groups rather than among individuals [10, 11, 13].

However, whether Hornsey et al.’s result [23] would readily extend to an intergroup/political apology context is not self-evident. First, no single item on the PAM was designed to measure attitudes toward intergroup apology. Second, and more importantly, previous studies on intergroup/political apologies revealed some systematic differences in the effects of interpersonal apologies and political apologies (see [25] for review). For example, although interpersonal apologies promote victim forgiveness and this effect is robust [22], intergroup/political apologies often fail to promote forgiveness [26, 27, 28]. Blatz and Philpot [25] thus point out the possibility that political apologies have effects on other dependent variables, such as impression of the apologizing parties. Moreover, typical political apologies contain more elements than typical interpersonal apologies: For example, political apologies tend to involve praise for both majority and minority groups [29]. Given such systematic differences in interpersonal and intergroup/political apologies, whether SDO has a comparable effect on intergroup apologies has yet to be investigated.

The current study aims to fill this gap, testing whether SDO is associated with unwillingness to issue an intergroup apology. Most apology-making settings involve one or more perpetrator(s) and victim(s) wherein the victim’s power, control, and status have been damaged by the perpetrator, while the perpetrator’s social evaluation may be degraded due to his or her wrongdoings. Accordingly, perpetrators may attempt to recover positive social evaluation by restoring a victim’s power, control, and status [30]. However, in an intergroup context, individuals high in SDO may not want to empower the victimized group, leading to loss of their group’s superiority over the victimized group. We predict, therefore, that individuals high in SDO will avoid apologizing to the group(s) that they have victimized.

This hypothesis was tested using an unpublished data set collected through a survey with a much broader scope than in this study. The survey included multiple choice questions and open-ended questions about Japanese respondents’ attitudes toward Japan’s apologies to other countries. In the survey, we measured respondents’ SDO and asked, in relation to past conflict among states, whether they would support government issued apologies. We also asked how uneasy they would feel if the Japanese government were to issue an apology.

The survey also included items addressing respondents’ self-perception of conservatism and militarism, which are used as control variables in the current study. Although there are no set of defining features of conservatism [31], characteristics commonly found among individuals high in conservatism include resistance to change and preference for inequality [32]. Previous studies have shown that SDO affects PAM even after controlling for conservatism [23]. The current study examines whether this finding extends to the intergroup context.

Further, militarism is associated with the belief that military power is necessary to defend national interest and, to a certain extent, the tendency to see the relationship among states as competitive. It is also associated with non-conciliatory attitudes toward other countries. Therefore, it is predicted that militarism has a negative correlation with apologies that aim toward reconciliation among states. This study tested whether the negative association between SDO and inclination toward intergroup apologies would remain intact after controlling for militarism.

## Materials and methods

The study was approved by the research ethics committee of Kobe University (school of law IRB, 29012). All participants signed the written informed consent at the beginning of the research.

The survey was conducted using a crowd-sourcing service in Japan. The total number of participants was 1639 (673 males, 899 females, and 67 unreported). The age of the participants ranged from 19 to 72 years, with a mean age of 38.2 years (SD = 10.7). After removing participants with at least one missing value on the variables of interest or responding as “I do not know”, 819 participants (385 males and 434 females) were retained for subsequent data analyses (S1 Dataset and S1 Table).

The variables that follow, presented in order of response, were used for the analyses (S1 and S2 Files). To assess Militarism, participants rated their level of support for the statement “in international politics, it is often necessary to use military power to protect national interests” on a four-point scale (1 = “strongly disagree” to 4 = “strongly agree”).

In examining Support for Governmental Apologies, participants rated their level of support for governmental apologies and expressions of regret directed toward the following recipients or concerning certain issues: (1) countries victimized by Japan during the colonial or occupation eras (Colonialism); (2) comfort women (i.e., women forced into sexual services) during WWII (Comfort Women); (3) the accident at the Fukushima Daiichi Nuclear Power Plant and ocean pollution after the Great East Japan Earthquake (Fukushima Daiichi); and (4) the massacre of Koreans in Japan by the militia, police, and military due to rumors scapegoating Koreans after the Great Kanto Earthquake (Kanto Massacre). These items were accompanied by a four-point scale (1 = “do not support at all” to 4 = “strongly support”).

To assess Resistance to Governmental Apologies, respondents reported how strongly they would feel resistance to the idea of the Japanese government issuing apologies and expressing regret. Specifically, participants rated their sense of resistance toward governmental apologies in general (General Resistance), apologies for atrocities during World War II (War Resistance), and the Fukushima Daiichi Nuclear Power Plant accident and ocean pollution (Fukushima Resistance). Participants rated General Resistance on a 4-point scale (1 = “do not feel any resistance” to 4 = “strongly feel resistance”) and the other two items on an 11-point scale (0 = “do not feel resistance” to 10 = “feel resistance”; 5 = “neutral”).

We used the Japanese version [19] of SDO_6_ [10, 11] to measure participants’ SDO. The scale comprises eight items pertaining to the endorsement of inequality among groups (e.g., “It’s probably a good thing that certain groups are at the top, and other groups are at the bottom; inferior groups should stay in their place”) and another eight items regarding the endorsement of equality among groups (e.g., “All groups should be given an equal chance in life; no one group should dominate in society”). The 16 items (after the latter eight item scores were reversed) were averaged to obtain a single SDO score (Cronbach’s α = .87).

In measuring Conservatism, participants rated their political attitude on an 11-point scale with two poles: 0 = “progressive” and 10 = “conservative,” with 5 as a choice indicating “neutral” [33]. Japanese political scientists use the word “progressive (*kakushin*)” in place of “left (*saha*)” or “liberal (*riberaru*)” to measure ideology in Japan [34, 35].

At the end of the survey, participants reported their birth year, gender, and education. Age was calculated by subtracting respondents’ self-reported birth year from 2018. As for gender, participants were asked to choose from three options: “male,” “female,” and “do not want to answer.” In the analysis, 1 was assigned to males and 0 was assigned to females; the gender of those who did not report their gender was treated was missing. In reporting education, participants chose one of the following options: “enrolled in either elementary, junior high, or high school, or withdrawn from any of them”; “withdrawn from high school, technical college, vocational school, or junior college”; “graduated from high school, technical college, vocational school, or junior college”; “enrolled in university or withdrawn from it”; “graduated from university”; “enrolled in graduate school or withdrawn from it”; and “graduated from graduate school.” These options were assigned a nominal scale (from 1 to 7 in this order).

## Results

Seven items regarding support for apologies and resistance to apologies were mutually correlated and showed a high level of internal consistency (α = .86; see S2 Table for the correlation matrix of the apology measures); thus, the seven item scores were averaged to a single score indicating participants’ willingness to apologize (General Apology: GA). The resistance scores were reverse coded. All seven items were standardized before the aggregation because the support scores and resistance scores were measured on different scales (namely 4-point and 11-point scales). This GA score showed significant and strong correlations with each of the seven individual items (*r*s > |.64|, see S3 Table). However, notice that the seven items consisted of conceptually distinctive groups: five war related items (Colonialism, Comfort Women, Kanto Massacre, General Resistance, and War Resistance) and two Fukushima related items (Fukushima Daiichi and Fukushima Resistance). In the main text, we report results associated with the three apology scores: GA, war apology (WA, α = .86), and Fukushima apology (FA, α = .79) (see S4 and S5 Tables for analyses of individual items).

Table 1 presents the correlation matrix of the variables of interest: SDO, Conservatism, Militarism, Gender, Age, Education, GA, WA, and FA. The reported *p* values were adjusted by the Holm method. Consistent with previous findings (e.g., [10, 11, 13]), SDO was positively correlated with Gender, Militarism, and Conservatism. More importantly, confirming our prediction, SDO was negatively correlated with all of three apology scores. However, Militarism and Conservatism were also negatively correlated with them.

**Table 1:** Mean, standard deviation and correlation of each variable.

	Mean	SD	Age	Education	SDO	Conservatism	Militarism	GA	WA	FA
Gender	0.47	0.5	0.19**	0.13**	0.19**	-0.02	0.18**	-0.1	-0.1*	-0.07
Age	39.58	11.22	1	0.05	-0.08	0.02	-0.06	-0.03	-0.08	0.04
Education	4.09	1.27		1	0.02	-0.01	0.03	-0.09	-0.08	-0.08
SDO	3.12	0.97			1	0.11*	0.33**	-0.36**	-0.35**	-0.3**
Conservatism	6.09	1.89				1	0.11*	-0.22**	-0.22**	-0.18**
Militarism	2.26	0.85					1	-0.39**	-0.4**	-0.29**
GA	0	0.74						1	0.95**	0.87**
WA	0	0.81							1	0.69**
FA	0	0.79								1

*p < .05

**p < .01, with adjusted p values

GA: General Apology, WA: War Apology, FA: Fukushima Apology

To eliminate the possibility that the negative correlation between SDO and apology scores was spurious due to the two variables’ association with either Militarism or Conservatism, we conducted a series of three multiple regression analyses including SDO, Militarism, and Conservatism, along with demographic variables (i.e., gender, age, education), as the predictor variables (see Table 2). The results showed that, for all three apology scores, the effects of SDO, Conservatism, and Militarism were negative and significant; that is, the negative association between SDO and the three apology scores remained intact even after controlling for the effects of Militarism, Conservatism, and other demographic variables. The same pattern was upheld when just the three predictor variables (SDO, Militarism, and Conservatism) were used for the analysis (S6 Table).

**Table 2:** Multiple regression models on apology.

	General Apology				War Apology				Fukushima Apology			
	Estimate	β		SE	Estimate	β		SE	Estimate	β		SE
SDO	-0.19	-0.25	**	0.02	-0.20	-0.24	**	0.03	-0.18	-0.22	**	0.03
Conservatism	-0.06	-0.16	**	0.01	-0.06	-0.15	**	0.01	-0.06	-0.13	**	0.01
militarism	-0.26	-0.30	**	0.03	-0.30	-0.32	**	0.03	-0.19	-0.21	**	0.03
male	0.03	0.02		0.05	0.04	0.03		0.05	0.02	0.01		0.05
Age	0.00	-0.06	*	0.00	-0.01	-0.12	**	0.00	0.00	0.01		0.00
education	-0.04	-0.08	*	0.02	-0.04	-0.07	*	0.02	-0.05	-0.08	*	0.02

*p < .05

**p < .01

## Discussion

This study demonstrated that SDO has a negative correlation with willingness to apologize in an intergroup context. Many studies have shown that individuals high in SDO tend to possess attitudes and behaviors (e.g., discriminatory and prejudicial) that are associated with factors that may escalate intergroup conflict. The current study provides additional insight into the relationship between SDO and conflict by showing that SDO is associated with reluctance to apologize. The effect of SDO on apologies remains significant after statistically controlling for the effects of Militarism and Conservatism. It should not be forgotten that conservatism indicates resistance to social change and preference for inequality, while militarism denotes hard-line attitudes toward interstate relationships. The independent effect of SDO, therefore, suggests that individuals high in SDO may avoid issuing apologies to other countries in order to prevent levelling of intergroup inequality. Issuing an apology is considered as an act to restore the victims’ power and status that has been damaged by the transgressors’ behavior [30]; thus, in the intergroup context, transgressors high in SDO might avoid apologies in order to prevent the victims’ status from approaching their own status.

We are aware that the current study is not without limitations. First, it is important to note that our measure of conservatism is dependent on participants’ self-placement on a 0–10 political ideology scale. It is also a single-item measure. Thus, some might doubt its reliability and validity. It is desirable to replicate the present study using other measures of conservatism [9]. Second, only control variables were included that could repress support for apologies in line with SDO (i.e., conservatism and militarism). In future studies, however, it would be necessary to include control variables that could promote support for intergroup apology. For instance, empathy fosters feelings toward apology [36] and it is negatively correlated to SDO [10]. Therefore, it would be important to test whether the factors fostering apology (such as empathy) and SDO have a meaningful and independent effect on the probability of (un)willingness to issue intergroup apologies.

## Supporting information

S1 Dataset Raw data. (XLSX)

S1 File Questionnaire in Japanese (original). (PDF)

S2 File Questionnaire in English (translated). (PDF)

S1 Table Descriptive statistics. (XLSX)

S2 Table Correlations between apology variables. (XLSX)

S3 Table Correlation matrix of War Apology, Fukushima Apology, and General Apology. (XLSX)

S4 Table Correlation matrix of individual apology items. (XLSX)

S5 Table Multiple regressions of individual apology items. (XLSX)

S6 Table Multiple regressions of War Apology, Fukushima Apology, and General Apology. (XLSX)
