# Supplementary material for: Social dominance orientation as an obstacle to intergroup apology
Source: PLoS One. 2019 Jan 25;14(1):e0211379. doi: 10.1371/journal.pone.0211379 (PMC6347393; doi:10.1371/journal.pone.0211379)
Supplement: S1 File — (PDF) [file pone.0211379.s002.pdf]

国際問題に関するアンケート
この度は、「国際問題に関するアンケート調査」にご参加くださり、誠にありがとうございます。

<u>調査の概要</u>

この調査では、いくつかの質問に回答していただきます。回答には、15分程度お時間をいただく見込みです。

この調査は、大学に所属する社会科学系の研究者が学術目的のために実施するものです。調査を通じて得られたデータは厳重に管理されるため、情報の漏えい等の心配はありません。調査の結果は研究目的のみで使用され、公表にあたっては個人が特定されることは決してありません。調査の結果は統計的に処理され、分析結果は論文等で発表されます。

以下の調査では、答えたくなくなった場合にはいつでも調査を終えることができます。また、答えたくない設問があった場合に、「わからない」という選択肢を選ぶこともできます。

ご意見やコメントは以下のメールで送信することが可能です。
experimentinsurvey@gmail.com

以上の内容をよくお読みいただきご理解いただいた上で、この調査に参加することに同意していただける場合は、「同意する」を選択した上で、右下の「＞＞」をクリックして、調査画面にお進みください。

<u>参加同意確認</u>

私は、この調査の趣旨を理解した上で、参加することに同意します。

○ 同意する (1)

○ 同意しない (2)

次の文章についてあなたはどう感じますか？　同意しますか。同意しませんか。
「国際政治では国家の国益を守るために、たびたび軍事力を用いることが必要になる」

- ○ 強く同意する
- ○ どちらかといえば同意する
- ○ どちらかといえば同意しない
- ○ 強く同意しない
- ○ わからない

これから国際問題にかかわる、さまざまな、謝罪やおわびについてお伺いします。あなたの考えにもっとも近い選択肢を選んでください。

日本は、過去に東アジアや東南アジアの諸国に対して、植民地支配や占領統治を行いました。日本政府がそういった植民地時代や占領統治時代にとった行為について、被害を受けた国に謝罪とおわびを行うことについて、どのくらい支持をしますか。

- ○ 強く支持する
- ○ どちらかと言えば支持する
- ○ どちらかと言えば支持しない
- ○ まったく支持しない
- ○ わからない

日本は、第二次世界大戦において、当時の軍の関与のもとに、従軍慰安婦として多くの女性の名誉と尊厳を深く傷つける行いをしました。日本政府がいわゆる従軍慰安婦問題について、被害者に謝罪とおわびを行うことについて、どのくらい支持をしますか。

- ○ 強く支持する
- ○ どちらかと言えば支持する
- ○ どちらかと言えば支持しない
- ○ まったく支持しない
- ○ わからない

日本は、2011年の東日本大震災において福島第一原発の事故を起こし、いまも微量ながら一定量の放射性物質が海洋流出する事態を招いています。日本政府が福島第一原発の事故と海洋汚染について、国際社会に謝罪とおわびを行うことについて、どのくらい支持をしますか。

- 強く支持する
- どちらかと言えば支持する
- どちらかと言えば支持しない
- まったく支持しない
- わからない

関東大震災の直後、「朝鮮人が井戸に毒を撒いた」などのデマがながされ、自警団・警察・軍人らが在日朝鮮人を虐殺する事件がありました。日本政府がこの事件について、謝罪とおわびを行うことについて、どのくらい支持をしますか。

- 強く支持する
- どちらかと言えば支持する
- どちらかと言えば支持しない
- まったく支持しない
- わからない

一般論として、日本政府が他国や他の国の人々に謝罪とおわびをすることについて、あなたは抵抗を感じますか。

- ◯ 強く抵抗を感じる
- ◯ どちらかと言えば抵抗を感じる
- ◯ どちらかと言えば抵抗を感じない
- ◯ 全く抵抗を感じない
- ◯ わからない

日本政府が第二次世界大戦の加害行為について謝罪とおわびを行うとしたら、あなた自身はそれにどのくらい心理的な抵抗がありますか。０が抵抗がない、５を中間、１０を抵抗があるという尺度であなたの考えにもっとも近い数字を選んでください。

- ◯ (抵抗がない)
- ◯ 1
- ◯ 2
- ◯ 3
- ◯ 4
- ◯ 中間
- ◯ 6
- ◯ 7
- ◯ 8
- ◯ 9
- ◯ (抵抗がある)
- ◯ わからない

日本政府が福島第一原発の放射能漏れ事故と海洋汚染について謝罪とおわびを行うとしたら、あなた自身はそれにどのくらい心理的な抵抗がありますか。０が抵抗がない、５を中間、１０を抵抗があるという尺度であなたの考えにもっとも近い数字を選んでください。

- ◯ (抵抗がない)
- ◯ 1
- ◯ 2
- ◯ 3
- ◯ 4
- ◯ 中間
- ◯ 6
- ◯ 7
- ◯ 8
- ◯ 9
- ◯ (抵抗がある)
- ◯ わからない

SDO 以下の文章を読んで、それぞれの主張にどれくらい同意するか、あるいは賛成するかを、1 から 7 の数字を選んで答えてください。あまり深く考えないで、直感的に答えてください。

（7 件法：1（全く同意しない／反対する）〜7（完全に同意する／賛成する））

1. ある種の人たちは他の集団の人たちよりも良い扱いを受けるに値する
2. 自分たちが欲しいものを手に入れるためには、他の集団に対して力をふるわなければならないこともある
3. ある種の人たちが他の集団と比べて人生のチャンスに恵まれているとしても、それはそれでかまわない
4. 人生で成功するためには、時として他の集団の人たちを踏み台にすることが必要だ
5. ある種の人たちの集団が身のほどをわきまえていたら、世の中の色々な問題は起こらないで済むだろう
6. ある種の人たちが上に立って、他の集団が下にいるのは、おそらくよいことだ
7. 劣った人たちの集団は、自分たちの立場をわきまえるべきである
8. 他の集団の人たちを現状に押しとどめておくべき場合がある
9. 全ての集団が平等になれたら良い
10. 私たちは集団間の平等を理想とすべきだ
11. すべての人たちの集団は人生のチャンスを等しく与えられるべきだ
12. 色々な集団が置かれた条件を等しくするために、私達はできるだけのことをすべきである
13. 私たちは社会的平等を目指すべきである
14. もし私たちが色んな集団をもっと平等に扱ってきたら、私たちの問題はもっと少なくなるだろう
15. 私たちは収入の平等をさらに目指すべきである
16. どんな集団も社会において支配的地位を独占するべきではない

政治に関して、ときどき、「保守、革新（＝リベラル）」という表現をすることがあります。
０が「革新」を意味し 10 が「保守」を意味するとします。たとえば、ちょうど中間くらいの立場のときには 5 を選んでいただく尺度になっています。

あなたご自身はどこに位置すると思いますか。この中の番号でお答えください。

- ○ 0 (革新)
- ○ 1
- ○ 2
- ○ 3
- ○ 4
- ○ 5 中間
- ○ 6
- ○ 7
- ○ 8
- ○ 9
- ○ 10 (保守)
- ○ 答えたくない

age あなたは、西暦何年生まれですか？

西暦 (65)

▼ 1945 年以前 (0) ... 2000 年以降 (55)

あなたの性別は、どちらですか？

- ○ 男性 (1)
- ○ 女性 (0)
- ○ 答えたくない (3)

あなたが最後に卒業した（あるいは現在、在学中の）学校は、次のうちどれでしょうか？

- ○ 小学校・中学校卒業または高校在学中・中退
- ○ 高校・高専・専門学校・短大在学中・中退
- ○ 高校・高専・専門学校・短大卒業
- ○ 大学在学中・中退
- ○ 大学卒業
- ○ 大学院在学中・中退
- ○ 大学院卒業
