# Supplementary material for: Social dominance orientation as an obstacle to intergroup apology
Source: PLoS One. 2019 Jan 25;14(1):e0211379. doi: 10.1371/journal.pone.0211379 (PMC6347393; doi:10.1371/journal.pone.0211379)
Supplement: S2 File — (PDF) [file pone.0211379.s003.pdf]

Questionnaire on international issues

Thank you very much for your interest in our “Survey on International Issues.”

Overview of the survey

In this survey, you will be asked to answer a set of questions. It will take about 15 minutes to complete the questionnaire.

This survey is conducted for academic purposes by a group of social scientists who belong to various universities. Your responses will be kept strictly confidential, and you do not need to worry about data leakage. The results of the survey will only be used for research purposes. When publishing the data, we will ensure your anonymity. The data from this survey will be statistically analyzed and reported in academic literature such as journals and working papers series.

If you do not want to answer further questions, you may stop participating at any time. Additionally, if you do not want to answer a particular question, you can choose the “do not know” option.

You may submit any comments and/or questions to the following e-mail address: ______@______

If you have read the terms listed above CAREFULLY and agree to participate in this survey, please select “Accept,” then click “>>” on the bottom right of your screen, and proceed to the survey screen.

Agreement confirmation

I confirm that I understand the purpose of this survey and agree to participate.

○ Agree (1)

○ Disagree (2)

How do you feel about the following sentence? Do you agree or disagree?
“In international politics, it is often necessary to use military power to protect national interests.”

- ◯ Strongly agree
- ◯ Somewhat agree
- ◯ Somewhat disagree
- ◯ Strongly disagree
- ◯ Do not know

Now, we would like to ask you about a variety of apologies involving international issues. Please choose the most appropriate option that reflects your thoughts.

Japan once placed East and Southeast Asian countries under colonial rule and occupation. To what extent do you support the Japanese government's apology to the victimized countries about its conduct during these colonial and occupational periods?

- ○ Strongly support
- ○ Somewhat support
- ○ Somewhat do not support
- ○ Do not support at all
- ○ Do not know

During World War II, Japan, with its military involvement, severely injured the honor and dignity of many women by forcing them to serve as comfort women. To what extent do you support the Japanese government's apology to the victims of the so-called comfort women issue?

- ○ Strongly support
- ○ Somewhat support
- ○ Somewhat do not support
- ○ Do not support at all
- ○ Do not know

Following the Great East Japan Earthquake of 2011, Japan had a critical nuclear accident at the Fukushima Daiichi nuclear power plant. A small but detectable amount of radioactive materials is still flowing into the ocean from the plant. To what extent do you support the Japanese government's apology to the international community about the accident and resultant marine pollution?

- ○ Strongly support
- ○ Somewhat support
- ○ Somewhat do not support
- ○ Do not support at all
- ○ Do not know

Immediately after the Great Kanto Earthquake, false rumors, such as "the Koreans are poisoning wells," became widespread and led Japanese vigilantes, police, and military personnel to commit the mass slaughter of Korean residents in Japan. To what extent do you support the Japanese government's apology regarding these actions?

- ○ Strongly support
- ○ Somewhat support
- ○ Somewhat do not support
- ○ Do not support at all
- ○ Do not know

In general, to what extent do you feel resistance to the idea that the Japanese government has apologized to the people and governments of other countries?

- ○ Strongly feel resistance
- ○ Somewhat feel resistance
- ○ Somewhat do not feel resistance
- ○ Do not feel any resistance
- ○ Do not know

To what extent do you feel resistance to the idea that the Japanese government should apologize for the transgressions it committed during the Second World War? Please choose the number that best describes your feeling: 0 means “do not feel resistance.” 5 means “neutral,” and 10 means “feel resistance.”

- ○ Do not feel resistance
- ○ 1
- ○ 2
- ○ 3
- ○ 4
- ○ Neutral
- ○ 6
- ○ 7
- ○ 8
- ○ 9
- ○ Feel resistance
- ○ I do not know

To what extent do you feel resistance to the idea that the Japanese government should apologize for the radioactivity leakage accident and resultant marine pollution of the Fukushima Daiichi nuclear power plant? Please choose the number that best describes your feeling: 0 means “do not feel resistance,” 5 means “neutral,” and 10 means “feel resistance.”

- ○ 0 Do not feel resistance
- ○ 1
- ○ 2
- ○ 3
- ○ 4
- ○ 5 Neutral
- ○ 6
- ○ 7
- ○ 8
- ○ 9
- ○ 10 Feel resistance
- ○ I do not know

Below are a series of statements with which you may either agree or disagree. For each statement, please indicate the degree of your agreement/disagreement by selecting the appropriate number from ‘1’ to ‘7’. Once again, remember that your first responses are usually the most accurate.
(Each item was rated on a 7-point scale ranging from 1 = “Strongly Disagree/Disapprove” to 7 = “Strongly Agree/Favor”)

1. Some groups of people are just more worthy than others.
2. In getting what your group wants, it is sometimes necessary to use force against other groups.
3. It’s OK if some groups have more of a chance in life than others.
4. To get ahead in life, it is sometimes necessary to step on other groups.
5. If certain groups of people stayed in their place, we would have fewer problems.
6. It’s probably a good thing that certain groups are at the top and other groups are at the bottom.
7. Inferior groups should stay in their place.
8. Sometimes other groups must be kept in their place.
9. It would be good if all groups could be equal.
10. Group equality should be our ideal.
11. All groups should be given an equal chance in life.
12. We should do what we can to equalize conditions for different groups.
13. We should increase social equality.
14. We would have fewer problems if we treated different groups more equally.
15. We should strive to make incomes more equal.
16. No one group should dominate in society.

With respect to politics, the expressions “conservative” and “progressive” (liberal) are sometimes used. Where would you locate your political view? Please select the most suitable number: 0 means “progressive” and 10 means “conservative.” For instance, if you feel like you are in the middle, selecting the number 5 would be an appropriate choice in this scale.

- ○ 0 Progressive
- ○ 1
- ○ 2
- ○ 3
- ○ 4
- ○ 5 Neutral
- ○ 6
- ○ 7
- ○ 8
- ○ 9
- ○ 10 Conservative
- ○ I do not want to answer

Please tell us your birth year.

▼ before 1945 (0) ... after 2000 (55)

Please tell us your gender.

- ○ Male (1)
- ○ Female (0)
- ○ Do not want to answer (3)

Which is the most appropriate choice to explain your educational status?

- ○ Enrolled in either elementary, junior high, or high school, or withdrawn from any of them
- ○ Withdrawn from high school, technical college, vocational school, or junior college
- ○ Graduated from high school, technical college, vocational school, or junior college
- ○ Enrolled in university or withdrawn from it
- ○ Graduated from university
- ○ Enrolled in graduate school or withdrawn from it
- ○ Graduated from graduate school
